# An Ebox Element in the Proximal *Gata4* Promoter Is Required for *Gata4* Expression *In Vivo*

**DOI:** 10.1371/journal.pone.0029038

**Published:** 2011-12-13

**Authors:** Alain Boulende Sab, Marie-France Bouchard, Mélanie Béland, Bruno Prud'homme, Ouliana Souchkova, Robert S. Viger, Nicolas Pilon

**Affiliations:** 1 Molecular Genetics of Development, Department of Biological Sciences and BioMed Research Center, Faculty of Sciences, University of Quebec at Montreal (UQAM), Montreal, Quebec, Canada; 2 Reproduction, Perinatal and Child Health, CHUQ Research Centre and Centre de Recherche en Biologie de la Reproduction (CRBR), Department of Obstetrics and Gynecology, Faculty of Medicine, Laval University, Quebec City, Quebec, Canada; Université Paris-Diderot, France

## Abstract

GATA4 is an essential transcription factor required for the development and function of multiple tissues, including a major role in gonadogenesis. Despite its crucial role, the molecular mechanisms that regulate *Gata4* expression *in vivo* remain poorly understood. We recently found that the *Gata4* gene is expressed as multiple transcripts with distinct 5′ origins. These co-expressed alternative transcripts are generated by different non-coding first exons with transcripts E1a and E1b being the most prominent. Moreover, we previously showed that an Ebox element, located in *Gata4* 5′ flanking sequences upstream of exon 1a, is important for the promoter activity of these sequences in cell lines. To confirm the importance of this element *in vivo*, we generated and characterized *Gata4* Ebox knockout mice. Quantitative PCR analyses realized on gonads, heart and liver at three developmental stages (embryonic, pre-pubertal and adult) revealed that the Ebox mutation leads to a robust and specific decrease (up to 89%) of *Gata4* E1a transcript expression in all tissues and stages examined. However, a detailed characterization of the gonads revealed normal morphology and GATA4 protein levels in these mutants. Our qPCR data further indicate that this outcome is most likely due to the presence of *Gata4* E1b mRNA, whose expression levels were not decreased by the Ebox mutation. In conclusion, our work clearly confirms the importance of the proximal Ebox element and suggests that adequate GATA4 protein expression is likely protected by a compensation mechanism between *Gata4* E1a and E1b transcripts operating at the translational level.

## Introduction

GATA family members (GATA1 to 6) are conserved among many species and display distinctive but overlapping spatio-temporal expression patterns [1], [2]. These proteins share a highly homologous zinc finger DNA binding domain and bind to a common consensus sequence motif (A/T)GATA(A/G) found in the promoter region of numerous genes. GATA members are subdivided in GATA1/2/3 and GATA4/5/6 subgroups. GATA1/2/3 factors are mostly but not exclusively involved in specification of hematopoietic lineages whereas GATA4/5/6 factors are involved in the development of mesodermal and endodermal-derived tissues, such as heart, gut, liver and gonads [2].

In the mouse, *Gata4* is known as the sole *Gata* gene expressed in somatic cells of the genital ridges around the time of sex determination at e10.5–11.5 [3], [4]. Strong *Gata4* expression then persists throughout male development and is maintained in adult Sertoli and Leydig cells. In the female, *Gata4* expression is slightly downregulated following sex determination but *Gata4* expression persists in postnatal and adult granulosa and thecal cells [3]. Although a number of GATA4 targets have first been identified via promoter studies in gonadal cell lines, a detailled analysis of the gonadal function of GATA4 *in vivo* has long been hampered by the early embryonic lethality of *Gata4*-null embryos at e9.5 [5], [6], [7]. This situation prompted the development of genetically-modified mouse models that circumvent early embryonic lethality and now provide clear evidence for a crucial and complex GATA4 role in the gonads. These partial and/or conditional *Gata4* loss-of-function models indicate that GATA4 is first required for normal gonad development in both sexes and then later for the proper function of the mature testis and the ovary [8], [9], [10], [11], [12].

Despite its pivotal role in gonadogenesis and many other developmental processes, relatively little is known about the factors and mechanisms that regulate *Gata4* expression. Using a RACE approach, we recently found that mouse, rat, and human *GATA4* genes are expressed as multiple transcripts that differ in their 5′origin owing to alternative usage of the first exon [13]. Two of these non-coding first exons, exon 1a and exon 1b, are conserved between species and are respectively located 3.5 and 31.5 kb upstream of the *Gata4* ORF start in exon 2. These two transcripts (named E1a and E1b) were found to be co-expressed in all GATA4-expressing tissues and both contribute to GATA4 protein synthesis [13]. In addition to alternative transcripts, it is known that transcriptional regulation of each *Gata* gene is complex, being controlled by multiple tissue-specific enhancers [14]. In this regard, a *Gata4* mesodermal enhancer sufficient to direct transgene expression in heart and liver was first identified in the zebrafish [15]. In the mouse, analyses of conserved non-coding sequences led to the identification of two distal enhancers located at 40 and 80 kb upstream of *Gata4* E1a transcriptional start site and sufficient to direct expression in the lateral mesoderm and a subset of endoderm derivatives respectively [16], [17]. The same approach recently led to the identification of a complementary endoderm enhancer located in intron 2 [18]. On the other hand, we previously reported that a 5 kb fragment of rat *Gata4* 5′ flanking sequences upstream of exon 1a is sufficient to direct reporter gene expression to previously unappreciated *Gata4* expression sites in a subset of cells from the inner cell mass of pre-implantation embryos as well as a subset of migratory neural crest cells [19]. Regarding gonadal expression, we also previously reported that the same 5-kb fragment controls reporter gene expression specifically in the testes and more precisely in Sertoli cells from embryonic to adult stages [20], [21]. A detailed analysis of this 5-kb fragment revealed an evolutionary conserved Ebox element (CACGTG) located near the transcriptional start site and we found this regulatory element to be critical for *Gata4* promoter activity *in vitro,* being bound by USF2 in gonadal cell lines [20], [22]. However, the importance of this Ebox motif *in vivo* is unknown.

Here we sought to determine the importance of the Ebox element in the regulation of *Gata4* expression *in vivo* by directly mutating the endogenous motif by homologous recombination in ES cells and derived mice containing the resulting *Gata4^EboxKO^* allele. Our data indicate that the Ebox motif is critical for the specific expression of the E1a transcript in all examined tissues and that the presence of E1b transcript can compensate the E1a loss and ensure appropriate levels of GATA4 protein.

## Materials and Methods

### Ethics Statement

Experiments involving mice were performed following Canadian Council of Animal Care (CCAC) guidelines for the care and manipulation of animals used in medical research. Protocols involving the manipulation of animals were approved by the institutional ethics committee of the University of Quebec at Montreal (comité institutionnel de protection des animaux (CIPA); Reference number 0511-R2-649-0512).

### Gene Targeting and Generation of Gata4^EboxKO-Neo^ and Gata4^EboxKO^ Mice

**Figure 1:**
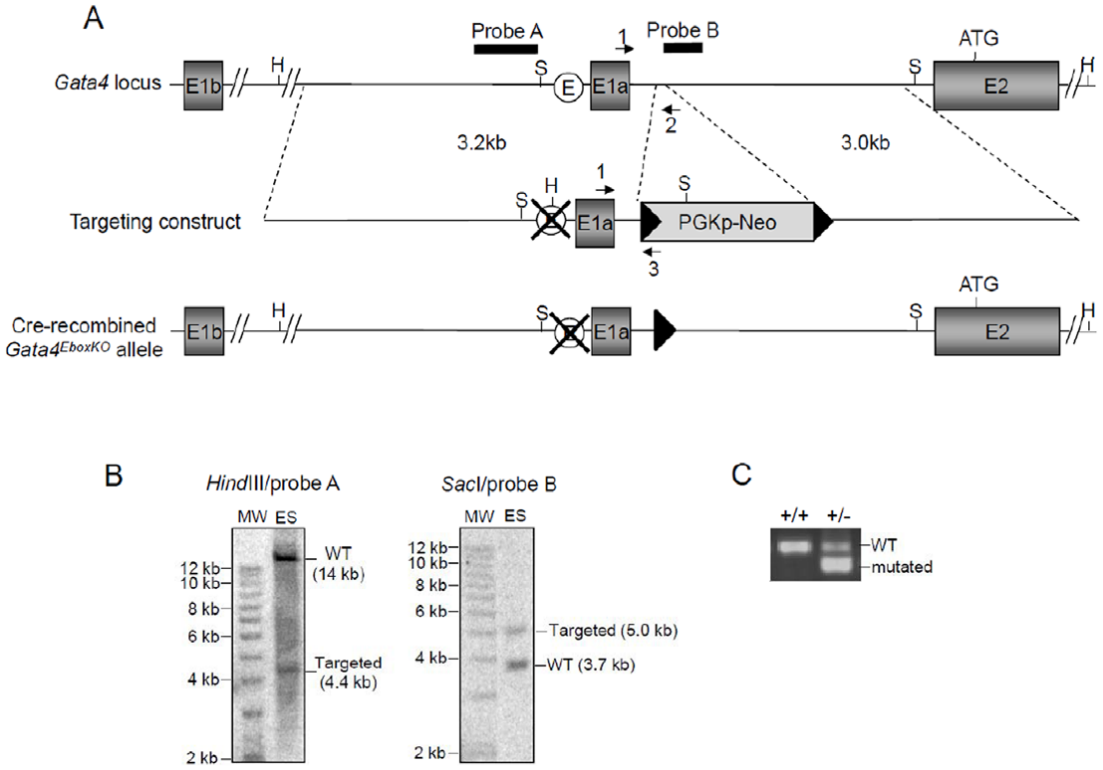
Targeting of the Ebox in the mouse *Gata4* promoter upstream of exon 1a. ***(A)*** Schematic representation of the *Gata4* locus, targeting vector and final Cre-recombined *Gata4* ^EboxKO^ allele. The targeting construct was designed to allow replacement of the *Gata4* Ebox (white circle) by a *Hind*III restriction site. A neomycin cassette flanked by LoxP sites (black triangles) was also included to allow G418-mediated selection in ES cells. Black arrows indicate oligonucleotide primers and solid black bars indicate the location of the probes used for Southern blotting. H, *Hind*III; S, *Sac*I. ***(B)*** Southern blot analyses confirming the predicted targeting event on both the 5′ and 3′ side for an ES cell clone used to generate chimeras. Genomic DNA was analyzed using the indicated combination of restriction endonuclease and probe. MW, 1 kb DNA ladder; ES, ES cell genomic DNA. ***(C)*** PCR analysis of tail genomic DNA from F1 offspring using the primers 1 and 2 to amplify the wild-type allele and primers 1 and 3 to identify the mutated allele.

As depicted in Fig. 1A, the targeting construct was designed to allow replacement of the endogenous *Gata4* Ebox element (CACGTG) by a *Hind*III restriction site (AAGCTT) and insertion of a PGKp-Neomycin cassette flanked by LoxP sites (Floxed Neo) in an 80 bp deletion in intron 1a-2. Sequences for the 5′ (3.2 kb) and 3′ (3.0 kb) homologous arms were obtained by PCR amplification of FVB/n mouse genomic DNA using the Advantage II DNA polymerase mix (Clontech). These PCR products were cloned into the pGEM-T plasmid (Promega, Madison, WI) and sequences were confirmed by sequencing. The Ebox element contained in the 5′ recombination arm was then converted to a *HindIII* site using the Quick Change Site-Directed Mutagenesis Kit according to the manufacturer's (Stratagene) instruction. The sequences of the oligonucleotides used for amplification of the recombination arms or site-directed mutagenesis are available upon request. The targeting construct was finalized by subcloning of the recombination arms on each side of a Floxed Neo cassette to allow G418-mediated selection in ES cells and subsequent Cre-mediated removal of the cassette if needed. R1 ES cells (Sv129 genetic background) were cultured on feeder cells under standard conditions as previously described [19], [23]. Cells were electroporated with 25 µg of linearized targeting vector and selected with G418 (225 µg/ml) for 7 days. Surviving clones were isolated and homologous recombination assessed by genomic Southern blot using *HindIII* restriction endonuclease and hybridization with a probe from the 5′ recombination arm as shown in Fig. 1B. Fidelity of the recombination event was then confirmed with *Sac*I restriction endonuclease and a probe from the 3′ recombination arm (Fig. 1B). Microinjection of targeted ES cells into blastocysts and production of chimeric mice was performed in accordance to standard procedures [24] by the microinjection service of IRCM (Institut de recherches cliniques de Montréal). These chimeras were then mated with C57BL/6 females and heterozygous animals bearing the *Gata4^EboxKO-Neo^* allele were identified by Southern blot analysis as described above. *Gata4^+/EboxKO^* (Neo cassette removed) animals were obtained from a cross between *Gata4^+/EboxKO-Neo^* and *Meox2*-Cre (C57BL/6 genetic background; kindly provided by Dr Annik Prat, IRCM) lines. Removal of the Neo cassette was confirmed by PCR.

*Gata4^EboxKO-Neo^* and *Gata4^EboxKO^* lines were maintained on a mixed Sv129-C57BL/6 genetic background and genotyping of both lines was performed by PCR using the following oligonucleotide primers:

- 1- *Gata4*-Fwd: 5′-GGAAACTGGAGCTGGCCAGGTAG-3′
- 2- *Gata4*-Rev: 5′-CACCCATCAGTTTTTGCTGCTAATC-3′
- 3- LoxP-Rev: 5′-TATACTAGAGCGGCCGGATCCAATC-3′

The wild-type allele was identified using primers 1 and 2 (234 bp) whereas the targeted allele was identified using primers 1 and 3 (169 bp) (Fig. 1A). For studies involving embryos, breeding mice were mated overnight and noon of the day that a vaginal plug was observed was designated as embryonic day (e) 0.5.

### Tissue Collection and Processing for qPCR analyses

*Gata4* expressing tissues (gonads, heart and liver) were collected at different developmental stages (e15.5, postnatal day (P)14 and adult mice) to assess expression of transcripts E1a and E1b by qPCR. Total RNA was isolated from mouse tissues using TRIzol reagent (Invitrogen, Burlington, Canada) in accordance to manufacturer's instruction. First strand cDNAs were synthesized from a 0.5 to 5 µg aliquot of the various RNAs using the Superscript II Reverse Transcriptase System (Invitrogen). Real time qPCR was performed using a LightCycler 1.5 instrument and the LightCycler FastStart DNA Master SYBR Green I kit (Roche Diagnostics Canada, Laval, Canada) according to the manufacturer's protocol. Primers used for qPCR are shown in Table 1. All qPCR runs were done using the following conditions: 10 min at 95°C followed by 35 cycles of denaturation (5 sec at 95°C), annealing (5 sec at 60°C), and extension (20 sec at 72°C) with a single acquisition of fluorescence levels at the end of each extension step. Each amplification was performed in duplicate using at least three different preparations of first-strand cDNAs prepared from each organ. The specificity of the amplified PCR products was confirmed by analysis of the melting curve and agarose gel electrophoresis. Differences in mRNA levels between samples were quantified using the standard curve method. DNA fragments containing E1a and E1b of the mouse *Gata4* gene were amplified by PCR and cloned into pGEM-T easy vector (Promega) to generate the E1a and E1b standards for preparing the dilution curves. A cloned fragment of the ribosomal gene *Rpl19* served as reference gene. Serial dilutions of the target and reference plasmids ranging from 0.1 fg/µl to 0.1 ng/µl were prepared in order to generate the standard curves. The amount of DNA for the target (E1a, E1b) and reference (Rpl19) in the unknown samples was calculated by the LightCycler software 3.5 (Roche Diagnostics Canada) using the respective dilution curves. Data are reported in arbitrary units as a ratio of the level of *Gata4* E1a or E1b mRNA variant in each sample to that of the *Rpl19* reference gene. For each time point, at least three animals per genotype were analyzed.

**Table 1:** Oligonucleotide primers used for qPCR.

Primer name	Sequence
*Gata4* E1a Forward	5′-TCCGCGGACTCACGGAGATC-3′
*Gata4* E1b Forward	5′-ACAGGCTGGAATCTCTGGGCCT-3′
*Gata4* E2 Reverse	5′-ACCAGAGCGGCTCCAGCGAA-3′
*RpL19* Forward	5′-CTGAAGGTCAAAGGGAATGTG-3′
*RpL19* Reverse	5′-GGACAGAGTCTTGATGATCTC-3′

### Histological analyses and immunohistochemistry

For histological analyses, testes and ovaries were harvested at the desired stage, fixed overnight in 4% paraformaldehyde and embedded in paraffin. Paraffin sections (4 µm) were stained with hematoxylin and eosin (H&E). Images were taken with a Leica DFC 495 camera mounted on a Leica DM 2000 microscope (Leica Microsystems Canada, Richmond Hill, Canada). For immunohistochemistry, Paraffin-embedded sections were deparaffinized, rehydrated, and treated with 10 mM citric acid in a microwave oven for 10–20 min to improve antibody penetration. Endogenous peroxidase activity was blocked with 3% hydrogen peroxidase, and nonspecific binding was prevented by using 10% horse serum. Sections were exposed overnight at 4°C to goat polyclonal anti-mouse GATA4 IgG antibody (sc-1237X, Santa Cruz Biotechnology, Santa Cruz, CA) diluted 1∶500 in blocking solution (PBS containing 0.1% BSA). Sections were counterstained with hematoxylin and mounted with Permount (Sigma-Aldrich, Oakville, Canada). For negative controls, primary antibody was omitted. Slides were analyzed with a Zeiss Akioskop II epifluorescence microscope (Carl Zeiss Canada, Toronto, Canada) connected to a digital camera (Spot RT Slider; Diagnostic Instruments, Sterling Heights, MI). For both histological and immunohistochemistry analyses, three animals per genotype and time point were examined.

### Whole-mount immunohistochemistry

Whole-mount immunohistochemistry was performed on e9.5 embryos as previously described [24]. Briefly, embryos were first collected in PBS and fixed in methanol: DMSO (4∶1) overnight at 4°C. The embryos were then bleached in methanol: DMSO: H2O2 (4∶1∶1) for 5 hr at room temperature, rehydrated for 30 min through 50% methanol, and finally PBS. Embryos were incubated twice in PBSMT (2% instant skim milk powder, 0.1% Triton X-100 in PBS) for 1 hr at room temperature, then with primary antibody goat anti-mouse GATA4 antibody (sc-1237X, Santa Cruz Biotechnology, CA, USA) diluted in PBSMT (1∶50) at 4°C overnight. Embryos were washed twice in PBSMT at 4°C and 3 times at room temperature for 1 hr each, followed by an overnight incubation at 4°C with horseradish peroxidase-conjugated rabbit anti-goat IgG (Santa Cruz Biotechnology, CA, USA) diluted in PBSMT (1∶500). Embryos were washed as described above with an additional final 20-min wash in PBT (0.2% BSA, 0.1% Triton X-100 in PBS) at room temperature. For the color reaction, embryos were pre-incubated with 0.3 mg/ml of 3, 30-diaminobenzidine tetrahydrochloride (Sigma-Aldrich) in PBT for 30 min at room temperature, followed by addition of H_2_O_2_ to 0.0003% and incubation at room temperature until a brown color develops. Embryos were then rinsed in PBT to stop the reaction, post-fixed overnight in 4% paraformaldehyde and cleared in glycerol:PBS (1∶1). Images were taken with a Leica DFC 495 camera mounted on a Leica M205 FA stereomicroscope (Leica Microsystems Canada).

### Statistical analysis

**Figure 2:**
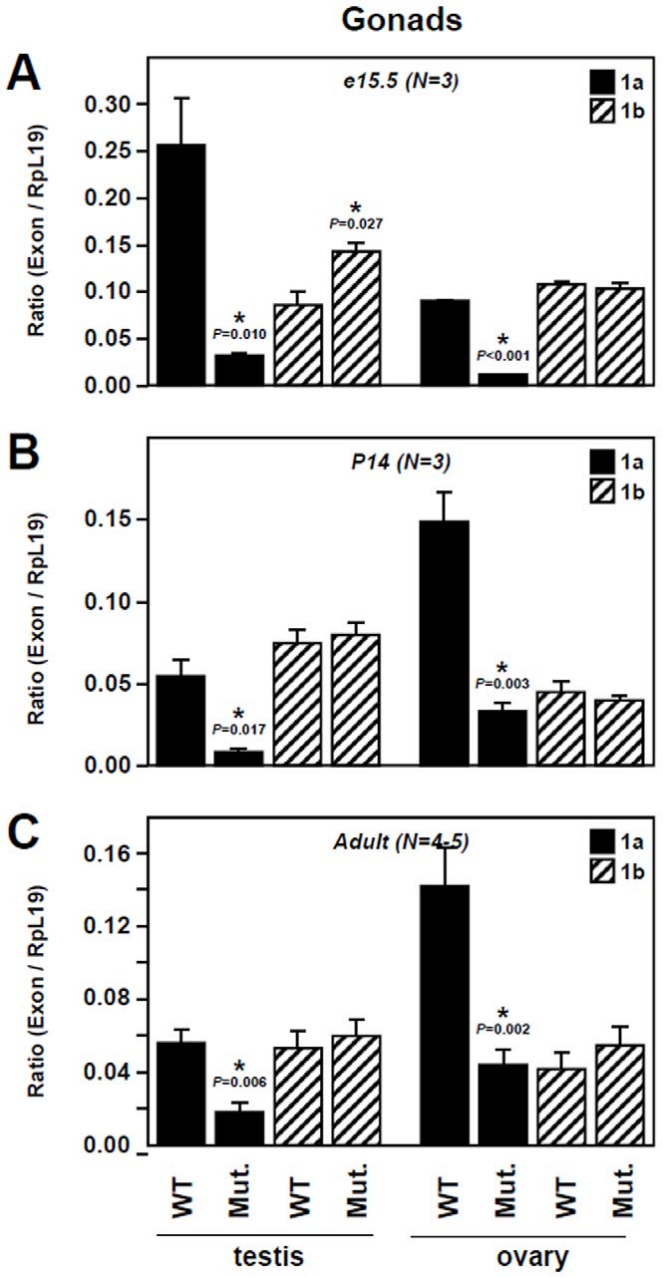
Comparison of gonadal E1a and E1b transcript levels between wild-type (WT) and *Gata4^EboxKO / EboxKO^* mutant animals. Quantitative PCR was used to assess *Gata4* E1a and E1b mRNA levels in mouse gonads at e15.5 (A), P14 (B) and adult (C) stages. Data are reported in arbitrary units as a ratio of the level of *Gata4* E1a or E1b mRNA variant to that of the *Rpl19* reference gene. *N* indicates the number of animals examined.

**Figure 3:**
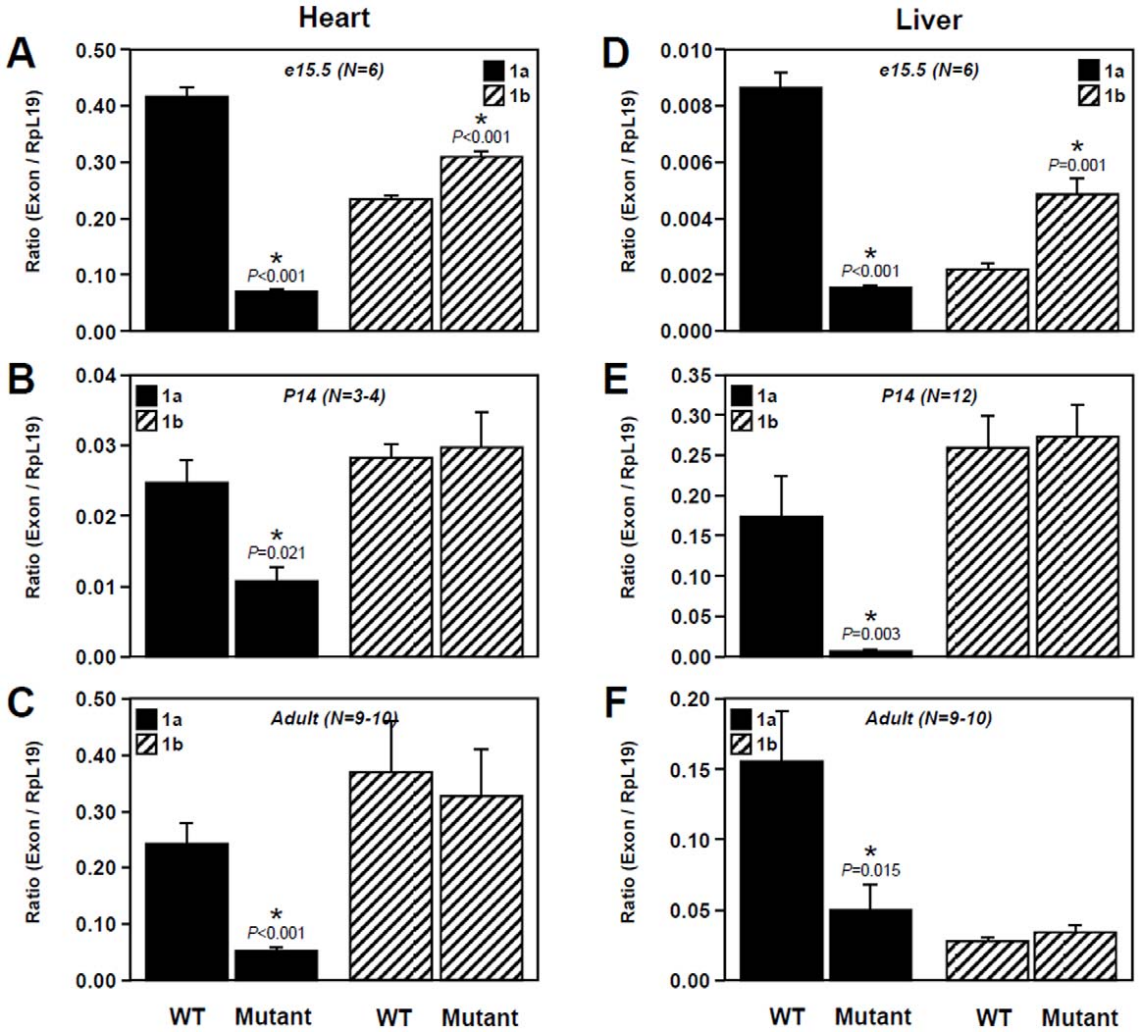
Comparison of extra-gonadal E1a and E1b transcript levels between wild-type (WT) and *Gata4^EboxKO/ EboxKO^* mutant animals. Quantitative PCR was used to assess *Gata4* E1a and E1b mRNA levels in the heart (A–C) and liver (D–F) at e15.5 (A, D), P14 (B, E) and adult (C, F) stages. Data are reported in arbitrary units as a ratio of the level of *Gata4* E1a or E1b mRNA variant to that of the *Rpl19* reference gene. *N* indicates the number of animals examined.

Comparisons of E1a and E1b transcript levels between wild-type and *Gata4^EboxKO/ EboxKO^* tissues (Figs. 2 and 3) were analyzed using Student's *t*-test; *P* < 0.05 was considered significant. All statistical analyses were done using SigmaStat 3.5 software (Systat Software Inc., San Jose, CA).

## Results

### Targeted mutation of the endogenous Gata4 Ebox element

Our previous analysis of the proximal 5′ sequences upstream of *Gata4* exon 1a, using luciferase assays and site-directed mutagenesis, demonstrated that an Ebox element is essential for promoter activity in several *Gata4*-expressing cell lines [20]. To confirm the importance of this motif *in vivo*, we generated a novel *Gata4* mutant allele in which the endogenous Ebox element (CACGTG) was replaced by a *Hind*III restriction site using homologous recombination in ES cells (Fig. 1A). The resulting allele containing the mutated Ebox was first designated *Gata4^EboxKO-Neo^* because of the persistence of a neomycin selection cassette flanked by LoxP sites (Floxed Neo) in intronic sequences between exon 1a and 2. Chimeras generated from *Gata4^+/EboxKO-Neo^* ES cells gave germline transmission and *Gata4^+/EboxKO-Neo^* animals were viable and fertile.

To confirm that the novel mutated motif is devoid of activity, we tested its functionality using an electrophoretic mobility shift assay (EMSA). In contrast to a wild-type probe, a probe bearing the mutated (*Hind*III) motif failed to compete the binding of USF1/2 proteins, demonstrating that the mutated element is indeed inactive (Fig. S1).

### The presence of a Neo selection cassette in intron 1a-2 leads to severe loss of GATA4 expression

Intercrosses of *Gata4^+/EboxKO-Neo^* animals were established but failed to produce viable homozygous offspring, suggesting embryonic lethality. Embryos from these matings were thus collected at e9.5, corresponding to the oldest stage at which non-resorbing *Gata4*-null embryos can be obtained [5], [6]. This analysis revealed that homozygous *Gata4^EboxKO-Neo^* embryos represented only 13.6% (32/233) of the total genotyped embryos. We also found that these homozygous *Gata4^EboxKO-Neo^* embryos displayed ventral morphology defects as well as an abnormal heart tube (Fig. S2A, B). Such non-mendelian ratio and morphogenesis defects are reminiscent of those previously reported in *Gata4*-nulls [5], [6] and suggest severe loss of GATA4 protein. In accordance with this, whole-mount immunohistochemistry revealed a drastic reduction of GATA4 protein levels in *Gata4^EboxKO-Neo/EboxKO-Neo^* e9.5 embryos (Fig. S2C, D).

To determine if this phenotype was due to the Ebox mutation or the insertion of the Neo cassette, we crossed *Gata4^+/EboxKO-Neo^* mice with *Meox2*-Cre mice [25] expressing the Cre recombinase in the epiblast in order to remove the floxed Neo cassette in all embryonic tissues (Fig. 1A). The resulting heterozygous as well as homozygous *Gata4^EboxKO^* mice did not display any obvious phenotype or altered sex ratios and were fertile (see Table 2). Thus, this indicates that the presence of the Neo cassette is responsible of the lethal phenotype of *Gata4^EboxKO-Neo/EboxKO-Neo^* embryos.

**Table 2:** Fertility tests for *Gata4^EboxKO/EboxKO^* mice.

Fertility Parameter	WT	Mutant
Breeding rate*	36.4% (12/33)	42% (13/31)
Pregnancy rate**	58.3% (7/12)	53.9% (7/13)
Average number of pups per litter	6.4	7

*Number of plugged females per total number of breeding females.

**Number of pregnant females per total number of plugged females.

### The Ebox motif is a specific and critical regulatory element of Gata4 E1a transcription *in vivo*

We have previously reported that *Gata4* is expressed as multiple transcripts with different 5′ ends encoded by alternative untranslated first exons and that two of these transcripts, E1a and E1b, are co-expressed in GATA4-expressing tissues from different species [13]. Therefore, to verify the impact of the Ebox mutation on *Gata4* expression, expression of transcripts E1a and E1b was assessed by qPCR in different tissues (gonads, heart and liver) and at different developmental stages (e15.5, P14 and adult).

In the gonads, this analysis first revealed sex- and stage-dependent differences in relative expression levels of each transcript in wild-type tissues. In the testis, transcript E1a is preferentially expressed at e15.5 (Fig. 2A) and then the level of each transcript becomes similar at the two week-old and adult stages (Fig. 2B, C). In the ovary, an inverted profile was observed with comparable expression levels of each transcript at e15.5 (Fig. 2A) followed by predominance of E1a transcript at the two week-old and adult stages (Fig. 2B, C). Interestingly, our data further indicate that, in homozygous *Gata4* ^EboxKO^ animals, a robust decrease of E1a transcript expression is observed in a sex- and stage-independent manner while E1b transcript expression remains essentially unchanged. This reduction is more profound in embryonic gonads (89%) (Fig. 2A) rather than at the two week-old and adult stages (80% and 70% respectively) (Fig. 2B, C). Of note, this analysis also revealed a slight but significant increase of E1b transcript levels in embryonic testes (Fig. 2A).

In extra-gonadal tissues, stage-dependent differences in relative expression levels of each transcript were also observed in wild-type tissues. In the heart, E1a transcript is predominantly expressed in embryos while E1b transcript is predominant in adult heart (Fig. 3A, C) and both transcripts are similarly expressed in two week-old animals (Fig. 3B). In the liver, E1a transcript is predominantly expressed in embryonic and adult tissues (Fig. 3D, F) while expression of the E1b transcript is predominant in two week-old animals (Fig. 3E). Again, in homozygous *Gata4* ^EboxKO^ animals, an important decrease of E1a transcript expression was observed in both the heart and liver. Much like the fetal testis (Fig. 2A), a slight but significant increase of E1b transcript was also specifically observed at the embryonic stage in heart and liver (Fig. 3A, D).

Taken together, these data clearly indicate that the Ebox element just upstream of *Gata4* exon 1a, is a key and specific regulatory element of *in vivo Gata4* E1a transcript expression in multiple GATA4-expressing tissues throughout development.

### A severe reduction of Gata4 E1a transcript expression is not associated with an overt gonadal phenotype

**Figure 4:**
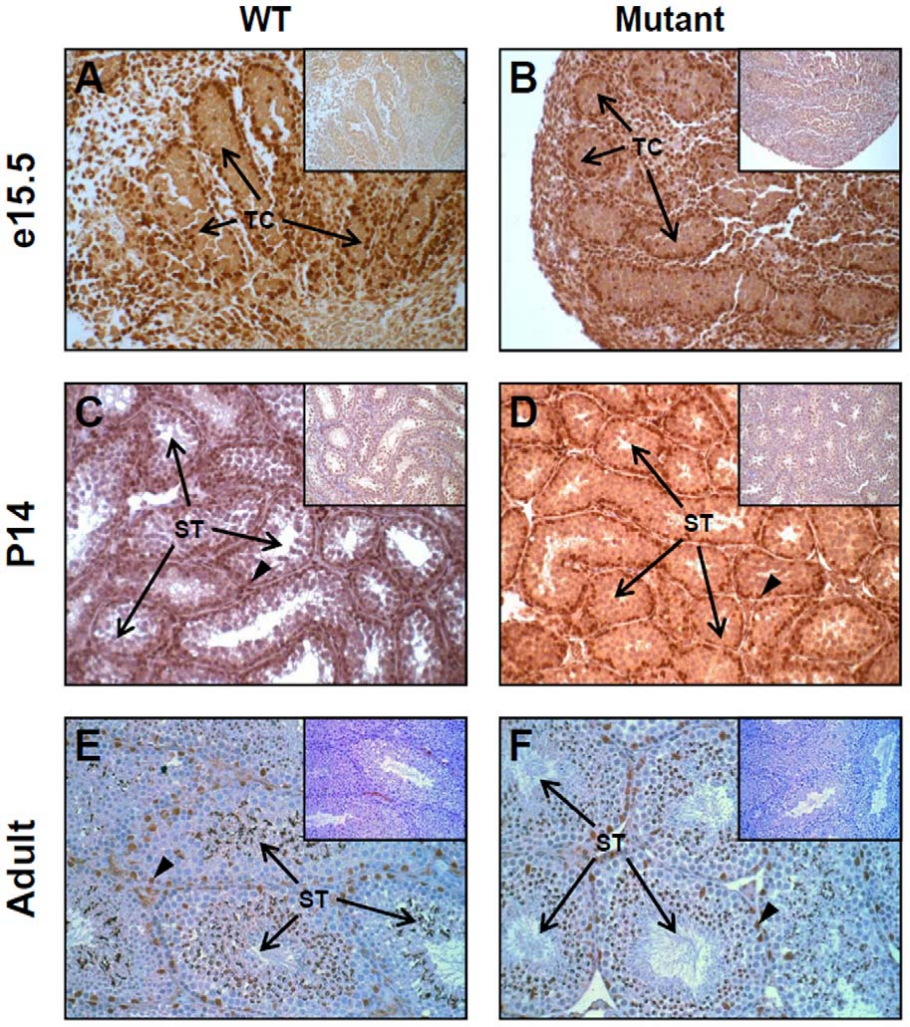
Analysis of endogenous GATA4 protein in *Gata4^EboxKO/ EboxKO^* mutant testes. GATA4 immunohistochemistry was performed on paraffin testis sections obtained from E15.5 embryos (A, B), two week-old (C, D) and adult mice (E, F). At each stage, note that no obvious differences in GATA4 protein levels are found between the wild-type (WT) and mutant testis. Arrowheads indicate the characteristic intense staining usually detected in Sertoli cells nuclei. Insets: control =  no primary antibody. Images were taken at 200x magnification. TC, testis cord; ST, seminiferous tubule.

**Figure 5:**
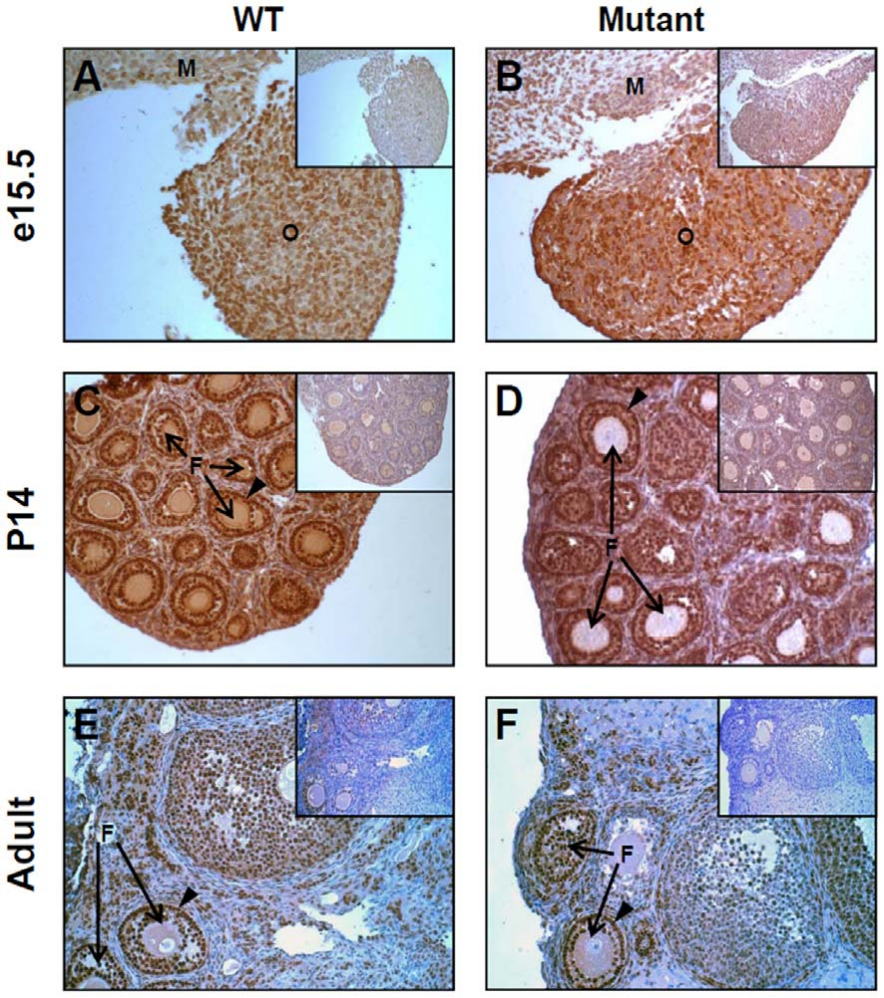
Analysis of endogenous GATA4 protein in *Gata4^EboxKO/ EboxKO^* mutant ovaries. GATA4 immunohistochemistry was performed on paraffin ovary sections obtained from E15.5 embryos (A, B), two week-old (C, D) and adult mice (E, F). At each stage, note that no obvious differences are found between the wild-type (WT) and mutant ovary. Arrowheads indicate the characteristic intense staining usually detected in granulosa cell nuclei. Insets: control =  no primary antibody. Images were taken at 200x magnification. O, ovary; M, mesonephros, F, follicle.

To assess the impact of E1a transcript reduction on gonad morphology, hematoxylin and eosin stained transverse sections of testes (Fig. S3) and ovaries (Fig. S4) were prepared from wild-type and homozygous *Gata4^EboxKO^* mice at the same developmental stages than those used for the qPCR analysis. This analysis revealed that *Gata4^EboxKO/EboxKO^* gonads from all stages do not display any gross histological defects. Although it was quite unexpected given the order of reduction of E1a transcript levels, such an outcome suggested that GATA4 protein levels were not significantly affected in *Gata4^EboxKO/EboxKO^* gonads. To verify this, GATA4 immunohistochemistry was performed on paraffin sections of wild-type and *Gata4^EboxKO/EboxKO^* gonads obtained at the same developmental stages. As shown in Figs. 4 and 5, and in accordance with a lack of gonadal phenotype, there was no obvious reduction of GATA4 protein levels in the mutant gonads. For both genotypes, GATA4 is strongly expressed in somatic cells of the testis and ovary at E15.5 (Fig. 4A–B, Fig. 5A–B), two week-old (Fig. 4C–D, Fig. 5C–D) and adult (Fig. 4E–F, Fig. 5E–F) stages.

On the other hand, we also hypothesized that a subtle change in GATA4 protein levels might have been missed by immunohistochemistry. To verify this possibility, we bred homozygous *Gata4^EboxKO^* mice with either *Gata4^+/EboxKO-Neo^* or *Gata4^+/-^* animals and analyzed the resulting *Gata4^EboxKO/EboxKO-Neo^* and *Gata4^EboxKO/-^* mice. This analysis revealed that, in both cases, animals appeared normal and were fertile (data not shown). Thus, even in combination with the null allele (50% reduction of GATA4 protein), the presence of the EboxKO allele does not lead to a functional drop in GATA4 protein levels.

## Discussion

The gene targeting approach used in this study led to the generation of a novel and unexpected severe hypomorphic *Gata4* allele. Indeed, we found that animals homozygous for the *Gata4^EboxKO-Neo^* allele, which retained the floxed PGKp-Neo selection cassette in intron 1a-2, recapitulate the *Gata4*-null phenotype and die around e9.5. Using Cre-mediated excision, we demonstrated that this phenotype is caused by the sole presence of the Neo cassette. There are several possible explanations for this outcome. The presence of these exogenous PGKp-Neo sequences might result in a general decrease in the total number of GATA4-encoding transcripts because of promoter competition from the PGKp sequences, abnormal splicing of exon 2 or defective intronic regulatory sequences. In support of the later, it is known that *Gata* tissue specific expression can be regulated via transcriptional enhancers located in introns [14] and a functional *Gata4* transcriptional enhancer has been recently identified in intron 2 [18]. However, the fact that a LoxP site still remains in the *Gata4^EboxKO^* allele following Cre-mediated recombination rules out the possibility that the phenotype might have been due to the fortuitously insertional disruption of a key *Gata4* regulatory element. On the other hand, we cannot rule out the possibility that insertion of the 3 kb Neo cassette might have isolated some regulatory elements crucial for expression of most of the *Gata4* mRNA variants. Taking this possibility into account, our targeting procedure led to the insertion of the Neo cassette in an 80 pb deletion in order to compensate for the final insertion of the LoxP site and surrounding plasmid sequences following Cre-mediated excision of the cassette; this strategy allowed preserving the exact same original spacing following Cre-mediated recombination. Irrespective of the exact hypomorphic mechanism, this novel *Gata4* ^EboxKO-Neo^ allele represents an interesting genetic tool for dissection of GATA4 functions if used in a setting allowing appropriate rescue of the ventral defects. Given that such defects in *Gata4*-nulls are caused by defective visceral endoderm, the *Transthyretin*-Cre mouse would represent a good model to use for rescue purposes [26], [27].

Although the mutation of the Ebox motif leads to a robust decrease of E1a transcript expression (between 70 and 89%), both tissue morphology and GATA4 protein levels appeared surprisingly unaffected in homozygous *Gata4^EboxKO^* gonads. This indicates that the *Gata4* E1b transcript variant, for which expression levels were either unaffected or slightly increased in homozygous *Gata4^EboxKO^* mutant tissues, is sufficient to ensure appropriate production of a normal and functional GATA4 protein. Such outcome is in total accordance with our prior work, where we showed by polysome analysis that both *Gata4* E1a and E1b transcripts are actively translated in mouse testes [13]. This observation also raises the intriguing possibility that, at least under certain circumstances, each transcript might be translated with different efficiency. In this regard, it is interesting to note that, among the several different ways by which translation can be regulated, the secondary structure of the 5′ UTR is known to have a profound influence on translation efficiency [28]. Indeed, it has been previously shown that loose structures at the 5′ end promotes whereas tight structures inhibits translation [29], [30]. In support of such a model for translation of the *Gata4* transcripts, E1a and E1b mRNAs precisely differ only by the extremity of their 5′ UTR and this appears to be enough for generating mRNA variants with different predicted secondary structures [13]. Further investigations will ultimately be required in order to understand this potential difference in transcript translation. For example, it would be particularly interesting to determine if E1b transcripts always exhibit preferential translation over the E1a variant or if this happens only when E1a levels are reduced.

In conclusion, this work clearly demonstrates that the Ebox motif of the proximal *Gata4* promoter is a key regulatory element of *Gata4* E1a transcript expression *in vivo.* More surprisingly, this work also underscored the fact that, even in the near absence of E1a transcripts, E1b transcripts can compensate and allow maintenance of normal and functional GATA4 protein levels. Considering the critical importance of GATA4 for the development and function of many organs, such a buffer mechanism is not altogether surprising.

## Supporting Information

Figure S1 **A mutated Ebox motif does not bind USF1/2 proteins.** Recombinant USF1 and USF2 proteins efficiently bind to a labeled probe containing the Ebox motif present in promoter sequences just upstream of exon 1a. Binding of USF1 and USF2 protein was competed by unlabeled wild-type probe (self) but not a probe containing the mutation (mut) used to generate the *Gata4* ^EboxKO^ allele.
(TIF)

Figure S2 **Embryonic lethality of homozygous** ***Gata4^EboxKO-Neo^*** **animals.** ***(A, B)*** In comparison to wild-type (A) littermates, surviving e9.5 *Gata4^EboxKO-Neo/EboxKO-Neo^* embryos (B) exhibit delayed development and severe ventral defects characterized by defective rostral-to-caudal and lateral-to-ventral folding as well as an abnormal heart tube. ***(C, D)*** Whole-mount immunohistochemistry on wild-type (C) and *Gata4^EboxKO-Neo/EboxKO-Neo^* (D) e9.5 embryos showing a drastic decrease of GATA4 protein in the mutants. Note that for immunohistochemistry, all age-matched embryos were processed and stained in parallel. Pictures were taken at 16X magnification.
(TIF)

Figure S3 **Histological analysis of** ***Gata4^EboxKO/EboxKO^*** **mutant testes.** Serial hematoxylin-and-eosin-stained transverse sections of testes obtained from the indicated developmental stages showing no significant histological differences between wild-type (WT) and mutant tissues. Images were taken at 100x magnification. TC, testis cord; ST, seminiferous tubule.
(TIF)

Figure S4 **Histological analysis of** ***Gata4^EboxKO/EboxKO^*** **mutant ovaries.** Serial hematoxylin-and-eosin-stained transverse sections of ovaries obtained from the indicated developmental stages showing no significant histological differences between wild-type (WT) and mutant tissues. Images were taken at 100x magnification. O, ovary; M, mesonephros, F, follicle.
(TIF)
