# Supplementary figures and images for: An Ebox Element in the Proximal Gata4 Promoter Is Required for Gata4 Expression In Vivo
Source: PLoS One. 2011 Dec 13;6(12):e29038. doi: 10.1371/journal.pone.0029038 (PMC3236771; doi:10.1371/journal.pone.0029038)

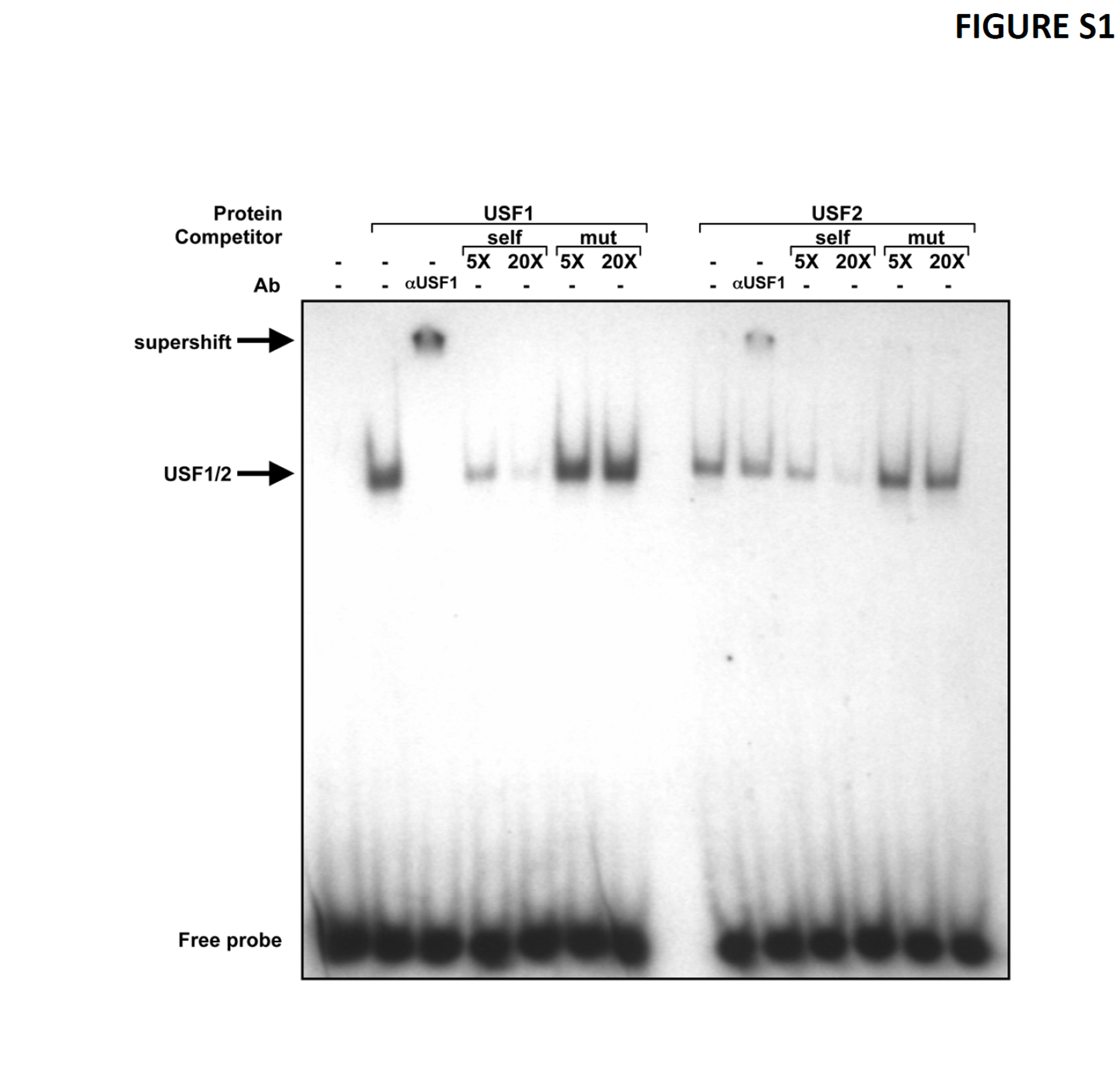

Supplement: Figure S1 — A mutated Ebox motif does not bind USF1/2 proteins. Recombinant USF1 and USF2 proteins efficiently bind to a labeled probe containing the Ebox motif present in promoter sequences just upstream of exon 1a. Binding of USF1 and USF2 protein was competed by unlabeled wild-type probe (self) but not a probe containing the mutation (mut) used to generate the Gata4 EboxKO allele. (TIF) [file pone.0029038.s001.tif]

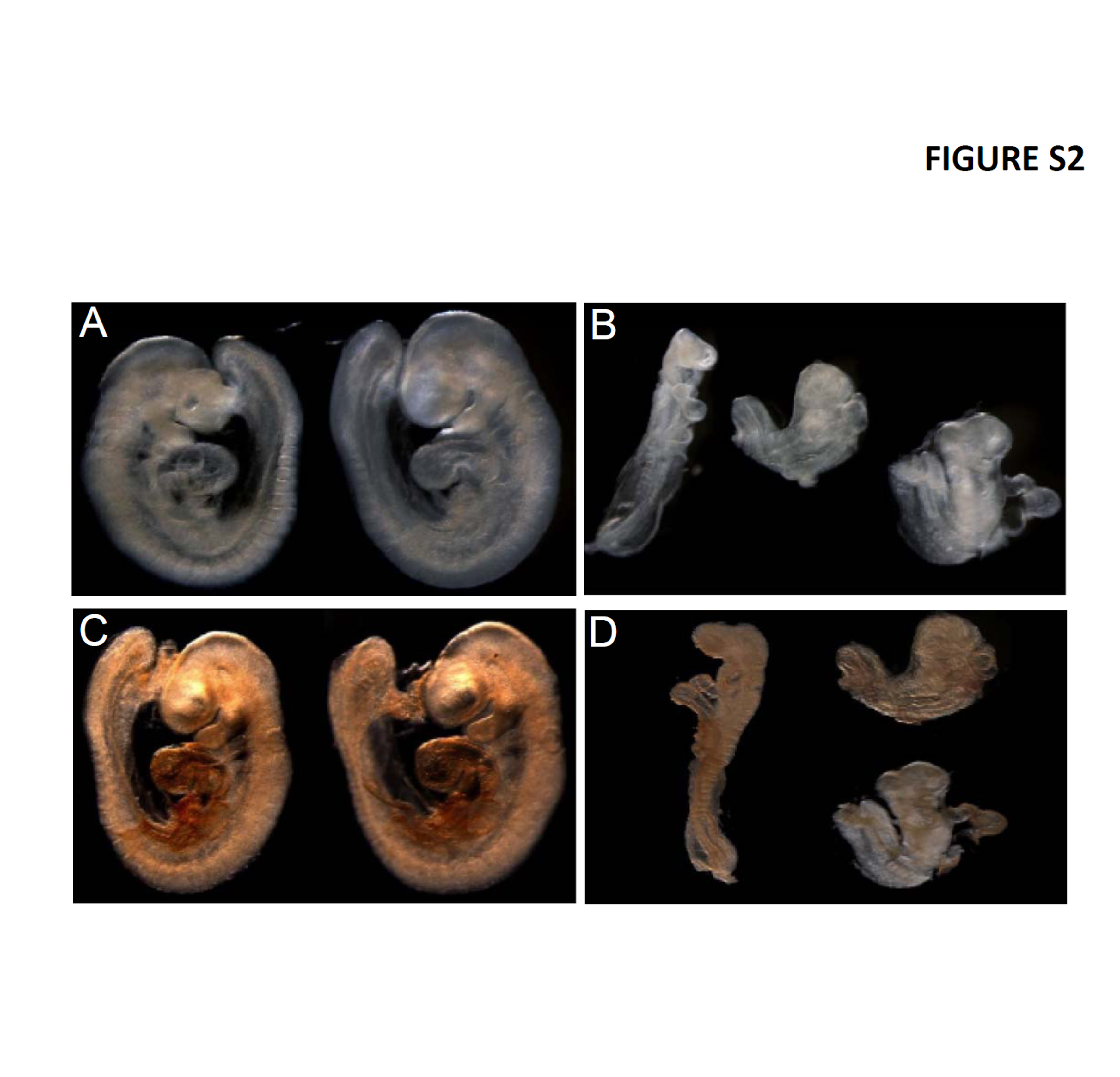

Supplement: Figure S2 — Embryonic lethality of homozygous Gata4EboxKO-Neo animals. (A, B) In comparison to wild-type (A) littermates, surviving e9.5 Gata4EboxKO-Neo/EboxKO-Neo embryos (B) exhibit delayed development and severe ventral defects characterized by defective rostral-to-caudal and lateral-to-ventral folding as well as an abnormal heart tube. (C, D) Whole-mount immunohistochemistry on wild-type (C) and Gata4EboxKO-Neo/EboxKO-Neo (D) e9.5 embryos showing a drastic decrease of GATA4 protein in the mutants. Note that for immunohistochemistry, all age-matched embryos were processed and stained in parallel. Pictures were taken at 16X magnification. (TIF) [file pone.0029038.s002.tif]

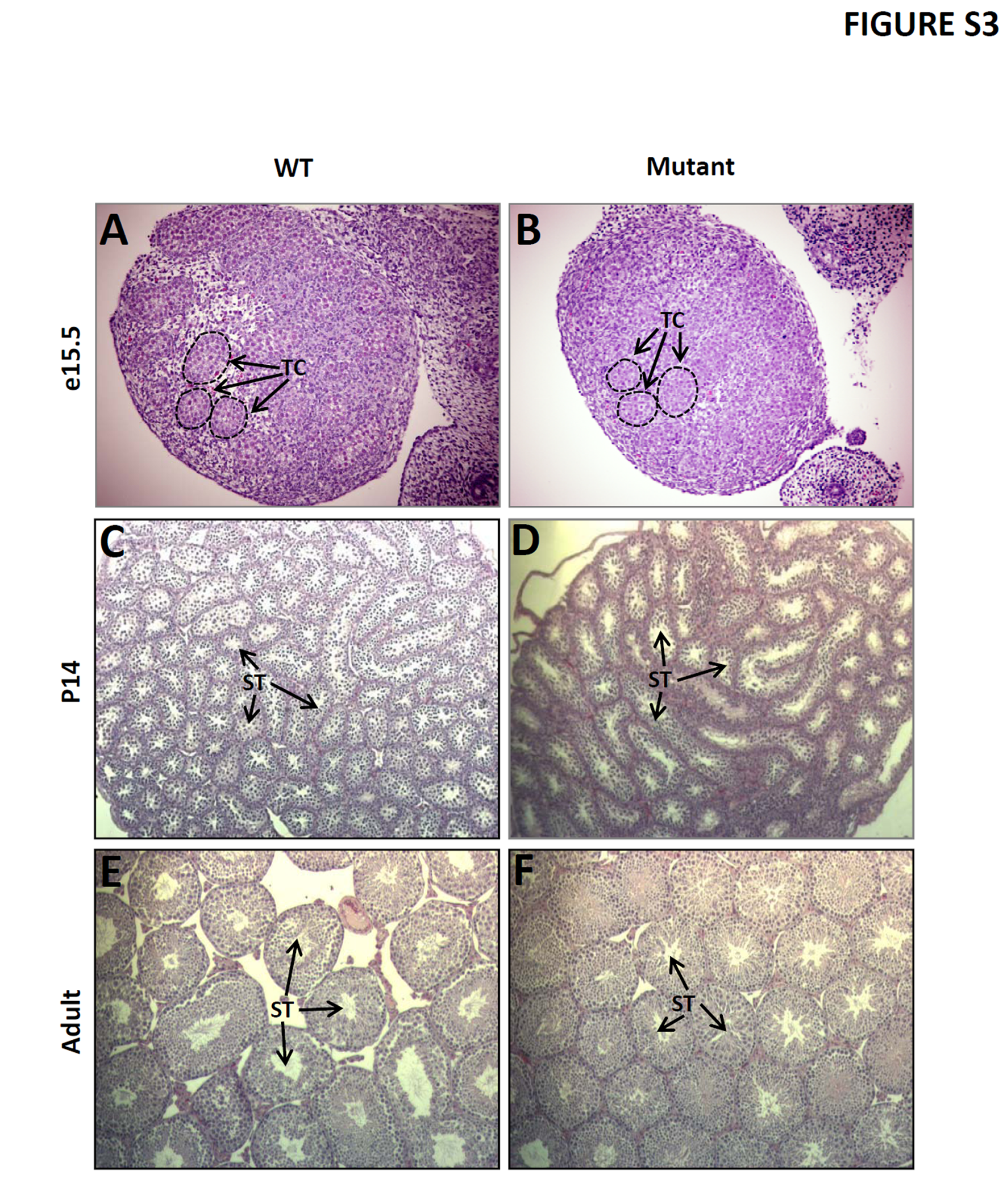

Supplement: Figure S3 — Histological analysis of Gata4EboxKO/EboxKO mutant testes. Serial hematoxylin-and-eosin-stained transverse sections of testes obtained from the indicated developmental stages showing no significant histological differences between wild-type (WT) and mutant tissues. Images were taken at 100x magnification. TC, testis cord; ST, seminiferous tubule. (TIF) [file pone.0029038.s003.tif]

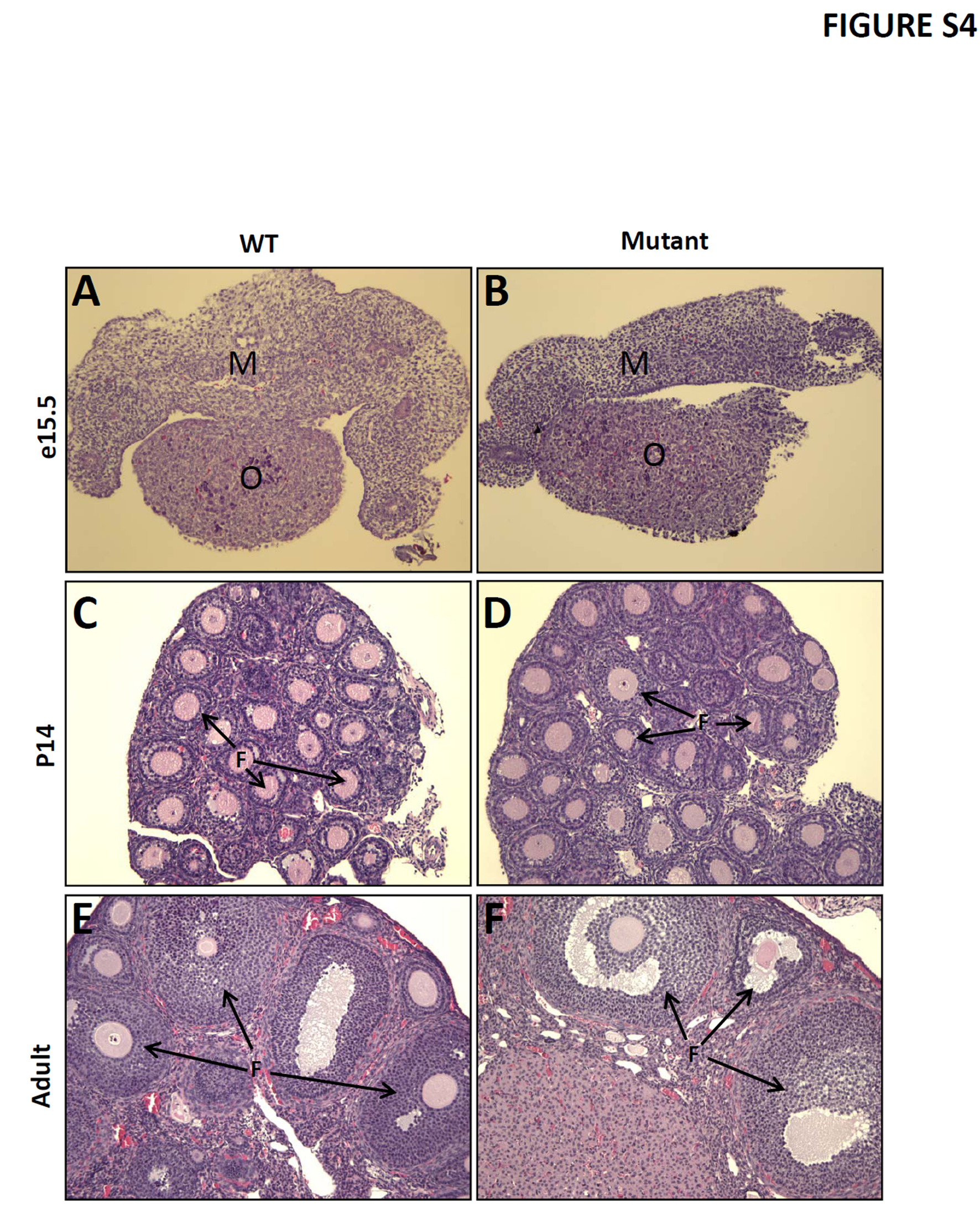

Supplement: Figure S4 — Histological analysis of Gata4EboxKO/EboxKO mutant ovaries. Serial hematoxylin-and-eosin-stained transverse sections of ovaries obtained from the indicated developmental stages showing no significant histological differences between wild-type (WT) and mutant tissues. Images were taken at 100x magnification. O, ovary; M, mesonephros, F, follicle. (TIF) [file pone.0029038.s004.tif]
